# Supplementary material for: Distinct Gene Expression Profiles in Colonic Organoids from Normotensive and the Spontaneously Hypertensive Rats
Source: Cells. 2021 Jun 17;10(6):1523. doi: 10.3390/cells10061523 (PMC8234507; doi:10.3390/cells10061523)

Figure S1 Unprocessed WBs used in Figure 2.

WKY (**W**) SHR (**R**) Colon (**C**) Organoid (**O**)

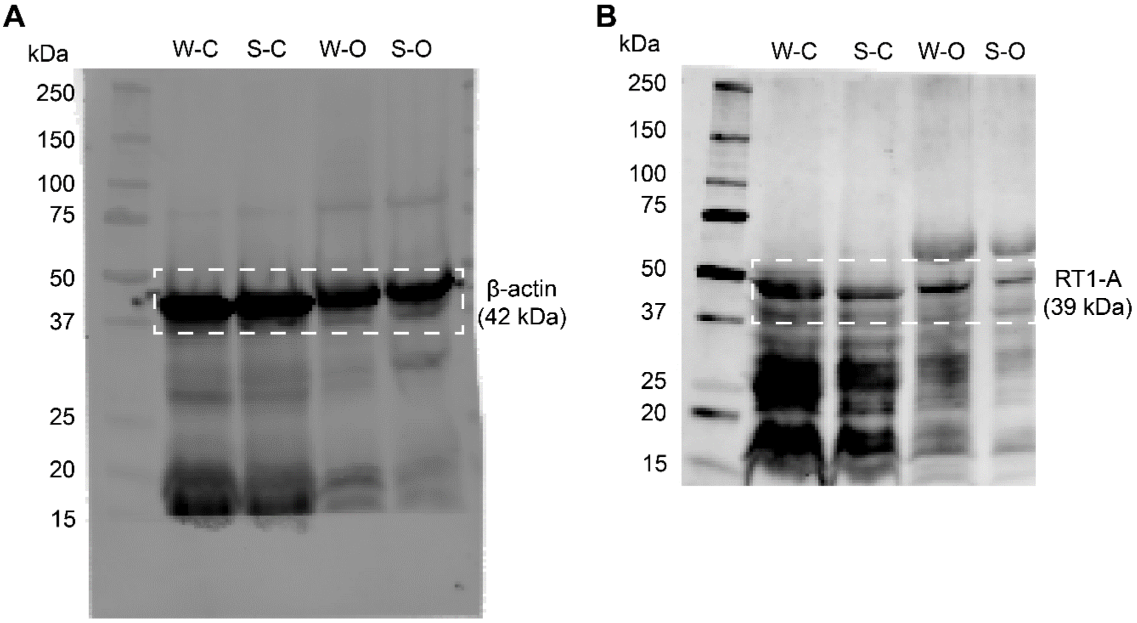

Figure S2 **Downregulation of RT1-A expression in colonic epithelium of SHR rats.** Fluorescent immunohistochemistry staining of RT1-A and Epcam in proximal colon sections of WKY and SHR rats, Epcam is a marker of epithelium (20× objective, scale bar: 50 μm).

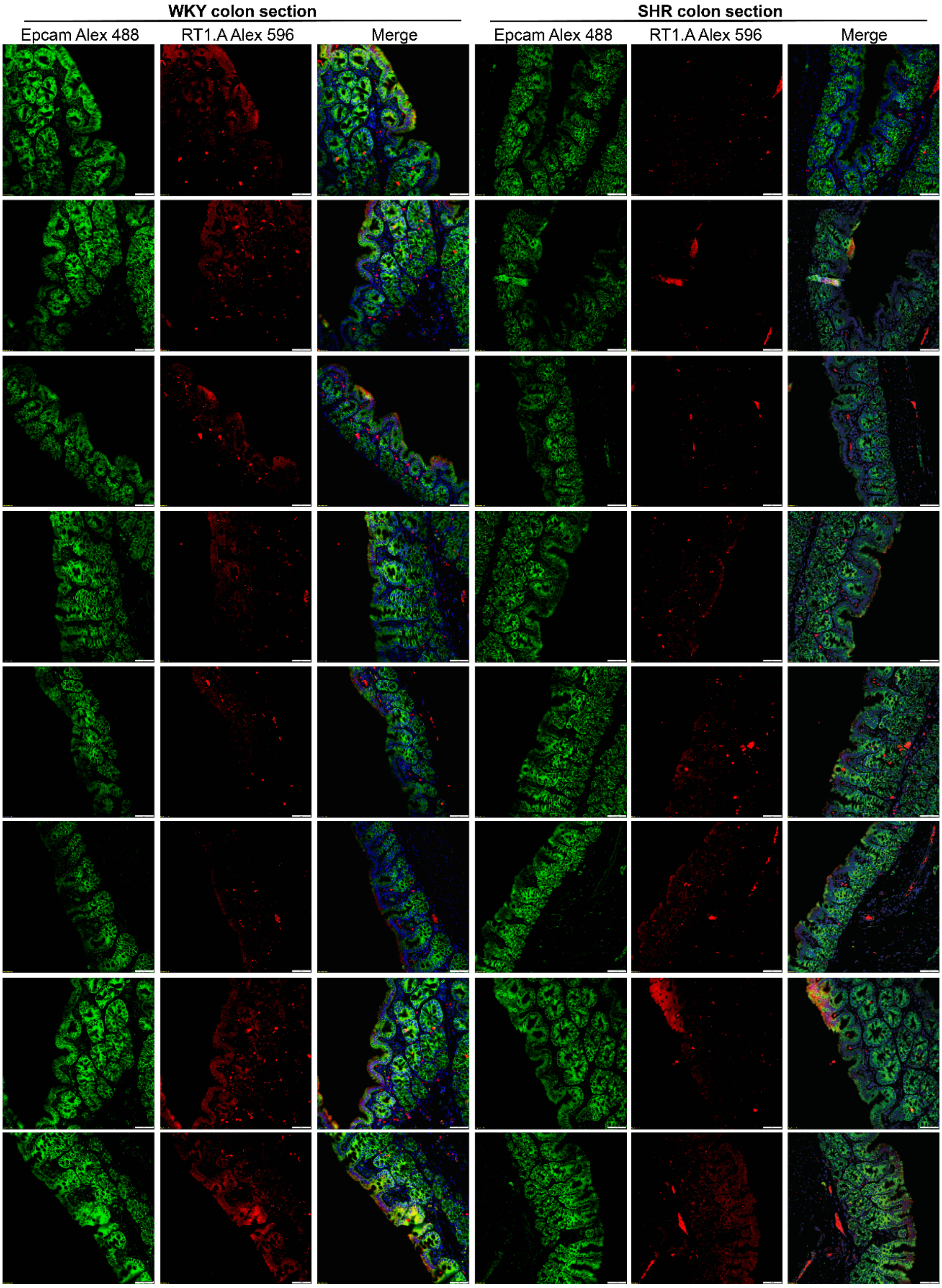

Supplement: Supplementary file 1 [file cells-10-01523-s001.zip › cells-1222882-supplementary.pdf]
